# Biomechanical effects of sitting with adjustable ischial and lumbar support on occupational low back pain: evaluation of sitting load and back muscle activity

**DOI:** 10.1186/1471-2474-10-17

**Published:** 2009-02-05

**Authors:** Mohsen Makhsous, Fang Lin, James Bankard, Ronald W Hendrix, Matthew Hepler, Joel Press

**Affiliations:** 1 Department of Physical Therapy and Human Movement Sciences, Northwestern University, Chicago, IL, USA; 2 Department of Physical Medicine & Rehabilitation, Northwestern University, Chicago, IL, USA; 3 Department of Orthopaedic Surgery, Northwestern University, Chicago, IL, USA; 4 Department of Radiology, Northwestern University, Chicago, IL, USA; 5 Department of Sensory Motor Performance Program, Rehabilitation Institute of Chicago, Chicago, IL, USA

## Abstract

**Background:**

Compared to standing posture, sitting decreases lumbar lordosis, increases low back muscle activity, disc pressure, and pressure on the ischium, which are associated with occupational LBP. A sitting device that reduces spinal load and low back muscle activities may help increase sitting comfort and reduce LBP risk. The objective of this study is to investigate the biomechanical effect of sitting with a reduced ischial support and an enhanced lumbar support (*Off-Loading*) on load, interface pressure and muscle activities.

**Methods:**

A laboratory test in low back pain (LBP) and asymptomatic subjects was designed to test the biomechanical effect of using the Off-Loading sitting posture. The load and interface pressure on seat and the backrest, and back muscle activities associated with usual and this *Off-Loading *posture were recorded and compared between the two postures.

**Results:**

Compared with *Normal *(sitting upright with full support of the seat and flat backrest) posture, sitting in *Off-Loading *posture significantly shifted the center of the force and the peak pressure on the seat anteriorly towards the thighs. It also significantly decreased the contact area on the seat and increased that on the backrest. It decreased the lumbar muscle activities significantly. These effects are similar in individuals with and without LBP.

**Conclusion:**

Sitting with reduced ischial support and enhanced lumbar support resulted in reduced sitting load on the lumbar spine and reduced the lumbar muscular activity, which may potentially reduce sitting-related LBP.

## Background

Occupational low back pain (LBP) is alarmingly common[1,2], with a 100 million workdays lost in the United States each year[3]. As the leading cause of disability in individuals less than 50 years old[4], LBP imposes a tremendous economic burden, with an estimated annual productivity lost of $28 billion in US[5]. Although the incidence of occupational back injury has been decreasing lately[6], the percentage of LBP among all the occupational back injury still is increasing[7] and occupational LBP remains the primary problem for US industry[6,7]. A number of occupational risk factors have been cited[8,9], prolonged sitting, in combination with awkward postures, has been confirmed to increase the likelihood of having LBP[10]. In fact, occupations which require prolonged sitting have a 3.2 relative risk (95% confidence interval) of LBP within the first year of employment[11]. Two frequently cited risk factors of LBP from occupational sitting are: 1), *prolonged static sitting *[8,9,12-20] and 2) *reduced lumbar lordosis *[8,15,21-26].

During sitting, upper body weight is carried mainly by the ischial tuberosities. Elevated pressures at the ischial tuberosities is intimately associated with elevated spinal loads[15]. Animal study provided evidence that sustained static load on the ligaments of the lumbar spine may result in paraspinal muscle spasm and hyperexcitability[13]. Furthermore, metabolite accumulation from static load may accelerate disc degeneration and herniation[15]. A lordotic lumbar spine has been considered a load-absorber in the manner of a spring[27] and it was found that the lumbar lordosis reduces intradiscal pressures[28] and transferring load to the posterior annulus and apophyseal joints[29]. These findings suggested that a lordosis in lumbar spine may have a protective effect on LBP[27].

Seating options, such as lumbar supports[22], forward tilted seat pans[22,30], and reclined seat/backrest have been assessed for the effect on reducing the spinal loading and paraspinal muscle activities during sitting. Although lumbar supports enhances lumbar lordosis, decrease intradiscal pressure[22,23], and may reduce paraspinal muscle hyperactivity, there is concern regarding the effectiveness of lumbar supports alone[22,31,32]. While these static seating designs have a positive effect in increasing lumbar lordosis or decrease pressure under the ischial tuberosity for the seated individual, they have limited effect in preventing pressure overload from *prolonged static *sitting. Therefore, periodic alternation between sitting and standing has been suggested to prevent the malignant effects of prolonged static sitting. Although it has an overall improved effect in LBP prevention than prolonged sitting [15-18], continuous spinal loading occurs in each position with minimal dynamic movement, providing minimal rest/change on muscular activation levels[9].

Makhsous et al. demonstrated that lumbar supports combined with an ischial release mechanism had a significant effect in decreasing ischial pressure and maintaining lumbar lordosis in asymptomatic subjects[31]. Therefore, we expected that this mechanism addressed some of the critical risk factors of LBP through enhanced lumbar support designed to maintain lumbar lordosis, and the reduced ischial pressure to reduce lumbar spinal load. The hypothesis of this study was that the *Off-Loading *posture will reduce the sitting load and lumbar muscular activity in LBP patients.

## Methods

Before participation, all participants gave written informed consent approved by the Northwestern University's IRB.

### Subjects

Twenty-five subjects with diagnosed LBP (15 female, 10 male; 41.3 ± 12.1 yrs; 72.1 ± 12.6 kg; 168.0 ± 8.5 cm; 25.4 ± 3.3 kg/m^2 ^for Body Mass Index (BMI)) and 10 asymptomatic subjects (2 female, 8 male; 30.5 ± 7.8 yrs; 80.6 ± 13.2 kg; 175.5 ± 9.1 cm; 26.0 ± 3.0 kg/m^2 ^for BMI) participated in the laboratory study.

LBP subjects were recruited and screened from the outpatient clinics of two of our investigators, MH and JP, who are physicians specializing in spinal disorders. Each patient underwent a thorough history, physical examination, chart review, and MRI imaging of the lumbosacral spine. Selection criteria included a diagnosis of chronic musculoskeletal LBP of greater than 3 months duration, with maximal pain located in the lumbosacral region. As the purpose of this study was to evaluate the biomechanical effects of sitting with adjustable ischial and lumbar support, to eliminate confounding factors, individuals with structural abnormalities of the spine (i.e., significant spondylosis, stenosis, significant disc space collapse, segmental instability, and/or scoliosis) and/or all individuals who were currently under aggressive treatment, such as surgery or epidural steroid injections, were excluded.

All asymptomatic subjects underwent a complete evaluation by the same physicians, including a history, physical examination, and standard radiographs of the lumbosacral spine, to screen for any history of LBP or spinal pathology.

### Study Chair Design

**Figure 1:**
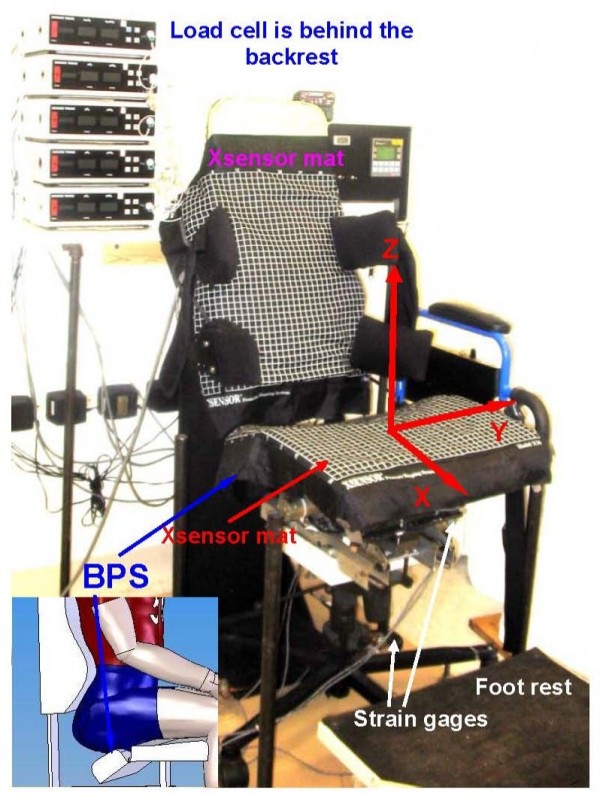
**Experimental setup for laboratory test**. The picture shows the actual experimental chair with Xsensor pressure mats on. It is shown in the *Off-Loading *configuration. The inset shows how the subject fit in the chair in an *Off-Loading *configuration, and the adjusting angle of the BPS.

An instrumented laboratory chair representing the proposed sitting concept[31] was utilized (Figure 1). The seat pan included a back part of the seat (BPS) which allowed inferior tilt down to 20° with respect to the front part of the seat (FPS). An attached motor allowed movement of the BPS. The seat pan was adjustable in depth, height, and width, to accommodate for varying body sizes. Both tilt angle of the BPS and seat depth were accurately recorded. The backrest-seat pan angle was set at 100°, with the seat pan parallel to the floor. An air bladder, with its pressure monitored by sensors, was embedded in the backrest, providing lumbar support adjustment in terms of height and protrusion. Automatic inflation/deflation of the lumbar support was achieved through an air pump. The air pressure threshold for the lumbar support was set between 7–8 KPa to avoid soft tissue injury. A Programmable Logic Controller (Vision 120, Unitronics, Israel) was used to control the motor and air pump, allowing the experimental chair to assume the following 2 sitting postures:

#### 1) Normal

Upright sitting using a regular flat seat pan and flat backrest.

#### 2) Off-Loading

Upright sitting with the BPS tilted downward 20° with respect to the FPS, and with protruded lumbar support.

### Testing Protocol

Each subject was initially asked to sit on the chair in the *Normal *posture, with their back flush against the backrest, and their ischia centered on the BPS. Both the backrest and seat pan were adjusted to accommodate for body habitus. The lumbar support was centered between the L2-L4 vertebral bodies, and the seat depth was adjusted to permit knee clearance. Seat height was adjusted to allow the feet of the subject to rest flush with the floor, with the knees flexed at 90°.

Simultaneous data collection started and lasted for 60 minutes. Posture change was regulated over a 10 minute period, i.e. the sitting configuration of the experimental chair was changed from one to another (*Normal *or *Off-Loading*) every 10 minutes. Therefore, three 10-minute recordings for *Normal *and 3 for *Off-Loading *postures were conducted for each subject.

### Pressure Distribution

Two pressure-mapping mats (Xsensor™ Pressure Mapping System, Calgary, Canada) were secured over the surface of the backrest and the seat pan. From the pressure recordings, total contact area (TCA), peak contact pressure (PP), and average pressure (AP) on both the backrest (TCA_BACK_, AP_BACK_) and the whole seat pan (TCA_SEAT-W_, AP_SEAT-W_) were calculated. Furthermore, the seat pan was divided into 3 horizontal regions (A: anterior, M: middle, and P: posterior), which allowed description of pressure distribution of each region (TCA_SEAT-A_, TCA_SEAT-M_, and, TCA_SEAT-P_), respectively. In addition, the location of PP along the anterior/posterior direction (PP_X_) on seat, the left/right direction (PP_Y_) on both the seat and backrest, and the superior/inferior direction (PP_Z_) on backrest were calculated.

### Sitting Load

Strain gauges were used to measure the load on the base of the chair. The center of load on the base (CL_BASE_) of the chair in horizontal plane and the magnitude of total load on the chair base (TL_BASE_) were obtained. The positive directions of the axes are shown in Figure 1.

### Backrest Load

Load on the backrest was measured along each axis using a six-axis load cell (JR3 Inc., Woodland, CA) mounted between the backrest frame and a rear post fixed on the floor. Total load on backrest (TL_BACK_) was determined by the magnitude of the resulting force.

### Paraspinal Muscle Activity

Activity of the paraspinal muscles at T5, T8, L2, and L5 levels was recorded using bi-polar surface EMG electrodes (Bagnoli-8, Delsys Inc, Boston, MA) with an amplification of 1000. EMG electrodes were placed at a distance of 3 cm from the spinous process on both sides. A reference electrode was placed at the spinous process of C7. After low-pass filtered at 230 Hz and then sampled into the computer at 500 Hz, each EMG signal was rectified and the envelope was obtained.

### Oswestry (2.0) and Roland-Morris LBP Disability Questionnaires

Baseline determination of LBP was obtained by the completion of the Roland Morris and Oswestry Low Back Pain Disability Questionnaires, which both were thought to perform as well as and better than other tools[33].

### Statistical Analysis

To eliminate the artifact caused by the movement of posture switching, data that fell in the posture switching stage (1 minute at the beginning and the end of each 10-minute interval) were discarded. This 2-minute period in which the data were discarded was considered as the wash-out phase for possible carry over effect for the order of posture adjustment, i.e. from *Normal *to *Off-Loading *or from *Off-Loading *to *Normal*. Then data for *Normal *and *Off-Loading *postures were first averaged over the three 8-minute intervals. When evaluating the posture effect, EMG and the interface pressure parameters, except the location of PP, were normalized to those in *Normal *posture for statistical analysis. To assess possible effect of the order of posture adjustment, i.e. the difference between the posture change from *Normal *to *Off-Loading *and from *Off-Loading *to *Normal*, all data were separated into these two categories and the order effect was tested using a one-way ANOVA. Then a two-way ANOVA with repeated measures over the posture effect was performed for each measurement to assess the within-subject posture effect (*Normal *vs. *Off-Loading*) and the between-subject group effect (Asymptomatic vs. LBP). To obtain the P values for each measurement on the posture effect, a one-way ANOVA was performed for each group. All statistical analysis was performed using the SPSS statistical software package (SPSS 16.0, SPSS, Inc, Chicago, IL) with a significance level at 0.05.

## Results

### Participant Profile

Tests results showed that there was significant difference on age (P = 0.039 using independent samples t-test) and gender distribution (P = 0.037 using Fisher's exact test) for two groups of participants. There was no significant difference on height, weight and BMI between the groups.

#### Oswestry (2.0) LBP Disability Questionnaire

The average Oswestry score for the 22 (3 were incomplete) valid questionnaires was 16.9 ± 9.6 out of 45. Seven participants did not complete section 8 (sex life). Thus, the average Oswestry score for the participants who fully completed the questionnaire was 18.4 ± 11.5 (N = 15) out of 50.

#### Roland-Morris LBP Disability Questionnaire

The average score for the 22 (3 were incomplete) valid Roland-Morris Questionnaires (RMQ) was 8.0 ± 4.4 out of 24. The highest RMQ score was 17 out of 24 for two of the subjects.

#### Pressure Distribution

**Figure 2:**
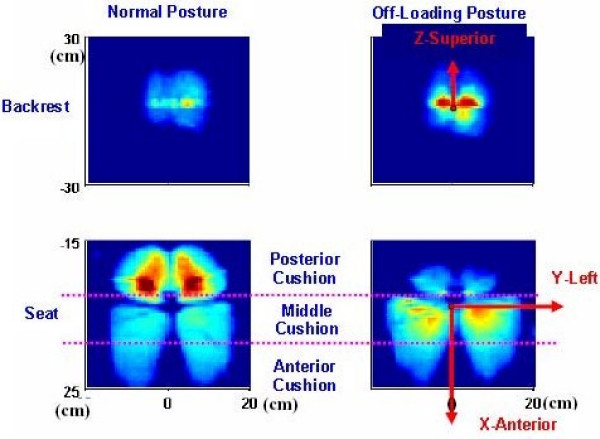
**Representative data of interface pressure from a subject**. Upper row: interface pressure between the backrest and the subject. Lower row: interface pressure between the seat cushion and the subject. Left column: recording in *Normal *posture. Right column: Recording in the *Off-Loading *posture. Recordings were done in the same trial.

A typical interface pressure recording of a subject sitting in both the *Normal *and *Off-Loading *postures is shown in Figure 2. In the *Normal *posture, the highest pressure loads are seen in the ischial (buttock) region of the subject.

#### Seat

The average (Mean ± SE) values of TCA, AP, and PP in *Normal *posture are listed for the whole cushion in Table 1. Among the 3 regions on the seat, the posterior region had the highest AP (Asymptomatic: 8.40 ± 3.37 KPa, LBP: 8.09 ± 0.33 KPa) and the highest PP (Asymptomatic: 24.62 ± 0.92 KPa, LBP: 22.88 ± 0.87 KPa), while posterior and the middle regions had similar contact area for both groups. The anterior region had the lowest AP for Asymptomatic and LBP groups.

**Table 1:** Summary of the average (Mean ± SE) values of Total Contact Area (TCA), average pressure (AP), and peak pressure (PP) in *Normal *posture on the whole seat and the backrest.

	**Asymptomatic (N = 10)**		**LBP (N = 25)**	
	
	**Seat**	**Backrest**	**Seat**	**Backrest**
**TCA (cm^2^)**	1225.5 ± 43.3	429.4 ± 37.0	1271.8 ± 36.2	434.9 ± 18.5
**AP (KPa)**	6.49 ± 0.28	4.53 ± 0.29	6.44 ± 0.17	4.48 ± 0.18
**PP (KPa)**	24.7 ± 0.9	22.3 ± 1.4	23.3 ± 0.8	21.4 ± 1.2

**Figure 3:**
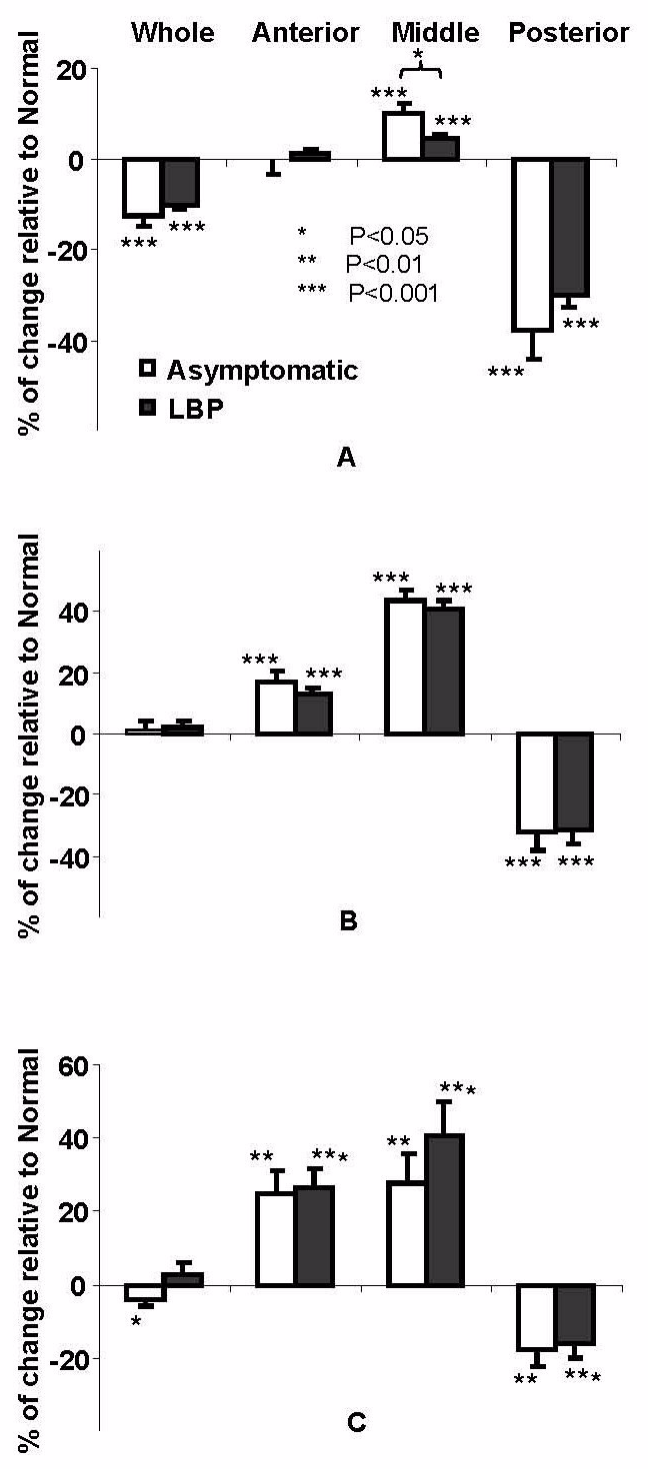
**Average changes (in percentage, Mean ± SE) of parameters of seat interface pressure induced by sitting posture change from *Normal *to *Off-Loading *for both Asymptomatic and LBP subjects**. A. Total contact area (TCA); B. Average pressure (AP); C. Peak pressure (PP).

The percentage change of the seat interface pressure parameters relative to the values of *Normal *posture are plotted in Figure 3. Looking at the whole seat cushion, changing from the *Normal *to the *Off-Loading *induced significant decreases in PP in Asymptomatic group and brought significant decreases in TCA for both groups. Significant decreases of AP, TCA, and PP_SEAT _were observed in posterior region. Conversely, for both groups, there were significant increases in AP and PP in both anterior and middle regions, and significant increase in TCA in middle region.

In comparison between the groups, significant difference was only seen in TCA in middle region, where the Asymptomatic group had a greater increase. For all other measurements, no significant interaction was found between the "group" effect and the "posture" effect.

#### Backrest

**Figure 4:**
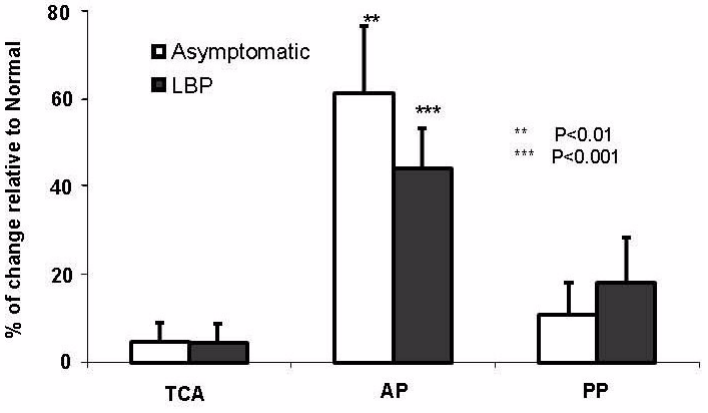
**Average changes (in percentage Mean ± SE) of parameters of backrest interface pressure (total contact area "TCA", average pressure "AP", peak pressure "PP", vertical location of PP "PP_Z_" and "horizontal location of PP "PP_Y_") induced by sitting posture change from *Normal *to *Off-Loading *posture for both Asymptomatic and LBP subjects**.

The average (Mean ± SE) values of TCA, AP, and PP in *Normal *posture are listed in Table 1. For both groups, changing from the *Normal *to the *Off-Loading *posture resulted in a significant increase in AP on the backrest (Figure 4). No significant interaction was found between the "group" effect and the "posture" effect and there was no significant group difference for the contact pressure on the backrest.

### Peak Pressure Location

#### Seat

For both groups, changing from the *Normal *to the *Off-Loading *posture, significantly (P < 0.001) translated PP_X _anteriorly towards the thighs.

#### Backrest

There was a significant superior shift of the peak pressure (PP_Z_) in LBP group (Figure 4).

No significant group difference was found for the location of peak pressure on the seat or the backrest.

### Sitting Load

#### Load on the chair base (Table 2)

**Table 2:** Load (CL: the center of load, TL: the total load) changes (Mean ± SE) on chair base induced by change from *Normal *to *Off-Loading*.

	**Asymptomatic (N = 10)**	**LBP (N = 25)**
**CL_BASE-anterior _(cm):**	8.86 ± 0.93	9.86 ± 0.56
***P*_*Normal*/*Off*-*loading*_**	<0.001	<0.001
***P*_*Group*_**	> 0.05	
		
**TL_BASE _(N):**	-32.47 ± 5.21	-40.60 ± 2.99
***P*_*Normal*/*Off*-*loading*_**	<0.001	<0.001
***P*_*Group*_**	> 0.05	

For both groups, changing from the *Normal *to *Off-Loading *posture significantly shifted the CL_BASE _anteriorly towards the thighs. Both groups had a significant decrease in TL_BASE_.

#### Load on backrest (Table 3)

**Table 3:** Load changes (Mean ± SE) on chair backrest in medial-lateral, inferior-superior and posterior-anterior directions, as well as the total load (TL) induced by change from *Normal *to *Off-Loading*.

	**Asymptomatic (N = 10)**	**LBP (N = 25)**
**Lateral load (N):**	-1.32 ± 0.86	-0.66 ± 0.67
***P*_*Normal*/*Off*-*loading*_**	>0.05	>0.05
***P*_*Group*_**	> 0.05	
		
**Superior load (N):**	-22.25 ± 1.44	-23.64 ± 1.96
***P*_*Normal*/*Off*-*loading*_**	**< 0.001**	**<0.001**
***P*_*Group*_**	> 0.05	
		
**Posterior load (N):**	66.79 ± 5.57	66.19 ± 3.42
***P*_*Normal*/*Off*-*loading*_**	**< 0.001**	**<0.001**
***P*_*Group*_**	> 0.05	
		
**TL_BACK _(N):**	69.66 ± 5.57	68.01 ± 4.16
***P*_*Normal*/*Off*-*loading*_**	**< 0.001**	**<0.001**
***P*_*Group*_**	> 0.05	

For both groups, changing from the *Normal *to the *Off-Loading *posture significantly increased total load on the backrest (TL_BACK_). Load born by the backrest significantly increased in posterior and inferior directions.

No significant group difference was found for the sitting load on the seat or the backrest.

#### Paraspinal Muscle Activity Study

As compared to the sessions of *Normal *posture, EMG levels at the Lumbar Levels are clearly decreased during the sessions of *Off-Loading *posture.

**Figure 5:**
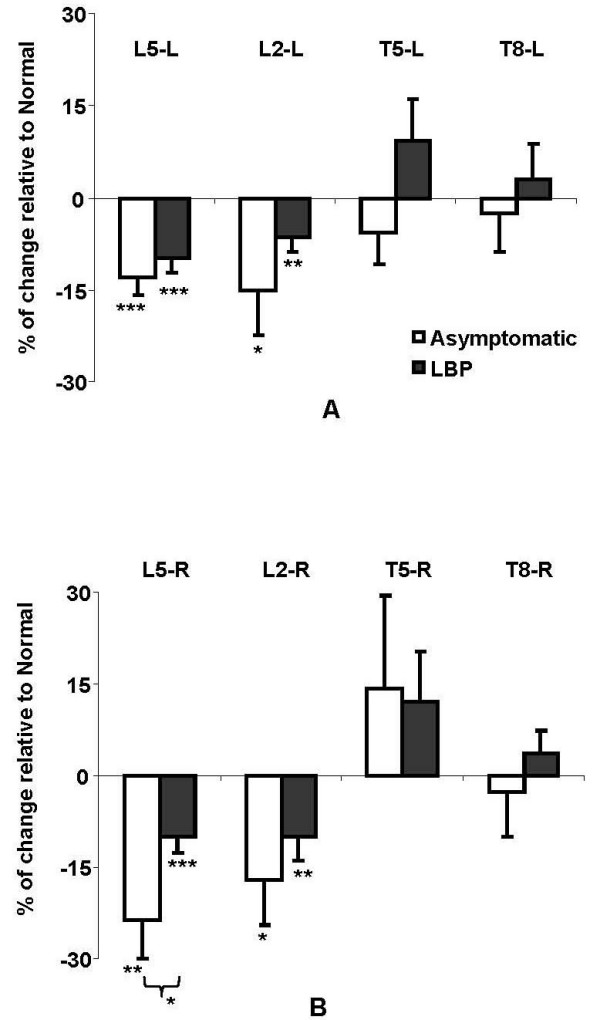
**Average changes (in percentage, Mean ± SE) of surface EMG from the back muscles induced by sitting posture change from *Normal *to *Off-Loading *posture for both Asymptomatic and LBP subjects**. A. Left side; B. Right side.

For both groups, shown in Figure 5, changing from the *Normal *to the *Off-Loading *posture significantly decreased EMG activity at both left and right sides of lumbar levels (L5, L2). The Asymptomatic group had a greater decrease in EMG lumbar paraspinal muscle activity (13% to 24%) than for the LBP group (6% to 10%); however, the significant group effect was seen only on right side at the L5 level. Also shown in Figure 5, there were some increases in the EMG in the thoracic region; however, they were not significant.

## Discussion

We reported previously[31] the beneficial biomechanical effects of sitting in the *Off-Loading *sitting posture in asymptomatic subjects. The purpose of this investigation was to determine whether the *Off-Loading *sitting posture was also beneficial to LBP individuals, and if any differences occurred between subjects with and without LBP. Our LBP target population was focused on occupational related LBP. Based on the Roland Morris and Oswestry Low Back Pain Questionnaires, our LBP group suffered from mechanical LBP with a moderate disability rating.

Callaghan, et al[9] showed that the sitting posture has significantly higher low back compressive loads than standing. As elevated ischial tuberosity pressures from sitting are intimately associated with elevated spinal loads, a seating device which can decrease ischial tuberosity pressures may help decrease and/or prevent LBP. Results of the current study demonstrate the beneficial effects of the *Off-Loading *sitting posture in both Asymptomatic and LBP subjects in improving overall pressure parameters, as both groups had a significant reduction of load on the ischial tuberosities, while redistributing load anteriorly towards the thighs, over a larger supporting surface. Furthermore, backrest load was increased, with the main component in the posterior direction. We believe that this increased posterior load on backrest has a beneficial effect in maintaining lumbar lordosis.

Many investigators have reported the negative effect of the seated posture associated with increased paraspinal muscle activity[8,21,23]. The seated posture results in sustained static load of lumbar viscoelastic tissues, resulting in spinal collagen micro-damage, paraspinal muscle spasm[12], and maybe a transient neuromuscular disorder[13]. It was[34] reported that significant mechanical loading of the spine is associated with LBP resulting from trunk muscle coactivation[34]. Thus, decreasing paraspinal muscle activity may also help minimize LBP. In this study, for both the Asymptomatic and LBP groups, the *Off-Loading *posture significantly decreased paraspinal muscle activity at the lumbar levels. These findings are similar to our previous study, in which we found decreased lumbar EMG levels in asymptomatic subjects with *Off-Loading *posture[31]. Our previous investigation has demonstrated that the *Off-Loading *posture helped rotate the pelvis forward, which may contribute to the decrease of the lumbar paraspinal muscular activity. As less paraspinal muscle effort is necessary to stabilize the spine, this may prevent muscle fatigue and improve overall comfort for the seated individuals.

Although the *Off-Loading *posture has the benefit of decreased lumbar paraspinal activity, we had concern that this sitting posture may possibly cause increased muscle activity at other spinal levels. However, our data demonstrate that the *Off-Loading *posture had no negative effect at the recorded thoracic levels.

We observed that, in the *Off-Loading *posture, the Asymptomatic group had a significantly greater increase of TCA on the middle seat, and a greater decrease in paraspinal muscle activity at the L5 and L2 levels, than those seen from LBP group. Although we are not certain as to the exact cause behind these differences, it is probably multifactorial, as it has been commonly observed that individuals with chronic LBP have difficulty in adopting a neutral posture of the lumbar spine, and that static balance might have been disturbed[35]. However, the mechanism underlying the differences should be further investigated.

The results from this investigation support our hypothesis that *Off-Loading *posture has a beneficial effect in pressure/load redistribution and lumbar paraspinal muscle activity in both asymptomatic and LBP subjects. In our previous pilot investigations[31], the *Off-Loading *posture also improved overall spinal alignment in a group of asymptomatic subjects. In yet another part of our study, we will investigate the effect of the *Off-Loading *posture in both LBP and asymptomatic subjects on lumbar lordosis. Furthermore, as prolonged static sitting is another major cause of LBP[8,9,12-20], our further study will also investigate the effect of *Alternate *sitting in a real working environment, on LBP subjects over a period of time.

One limitation of the study was the difference for the sample size for the two tested groups. The finding that there was no difference between the groups for most of the measurements should be generalized with caution.

## Conclusion

The *Off-Loading *sitting posture, defined as a released ischial support and an enhanced lumbar support, has been found to significantly redistribute the sitting load passing through ischial tuberosities to the lumbar spine, and reducing lumbar paraspinal muscle activity in both asymptomatic and LBP subjects. It is concluded that this *Off-Loading *sitting posture might be beneficial to people whose profession requires prolonged sitting.

## Competing interests

The authors declare that they have no competing interests.

## Authors' contributions

MM and FL came up with the original idea of the study, designed the entire research, and analyzed the data. JB implemented and coordinated the actual laboratory data collection and data processing. RWH, MH, and JP collected clinical data, ensured the clinical significance and relevance of the study, and helped interpret the results. While MM and FL were primarily responsible for writing the manuscript, all authors contributed to the writing from his point of view and expertise.

## Pre-publication history

The pre-publication history for this paper can be accessed here:


